# EXPLORING INFECTION PREVENTION AND CONTROL MEASURES USED BY TRADITIONAL PRACTITIONERS IN RWANDA

**DOI:** 10.21010/Ajidv19i2S.8

**Published:** 2025-10-17

**Authors:** KUBWIMANA Liberee, DUSINGIZE Marie Immaculee, MUTATSINEZA Gislaine, INGABIRE Peace, NKURUNZIZA Fred, SHIMELASH Natnael, WONG Rex

**Affiliations:** 1 School of Medicine, University of Global Health Equity, Kigali, KG 7 Ave, Rwanda; 2 Head, East Africa Biodesign Fellowship Program, University of Global Health Equity, Kigali, KG 7 Ave, Rwanda; 3 Bill and Joyce Cummings Institute of Global Health, Director of MGHD, Chair of Innovation Center, University of Global Health Equity, Kigali, KG 7 Ave, Rwanda; 4 Director of MGHD, Chair of Innovation Center, University of Global Health Equity, Kigali, KG 7 Ave, Rwanda

**Keywords:** Traditional medicine, Traditional Practitioners, infection prevention, infection control practices, Healthcare, Medicine in Rwanda

## Abstract

**Background:**

Traditional Practitioners (TPs) serve as primary healthcare providers in Rwanda, offering culturally relevant healing methods. Despite their importance, there’s a lack of understanding regarding their infection prevention and control (IPC) practices.

**Materials and Methods:**

The study conducted in-depth interviews with Traditional Practitioners (TPs) in four Rwandan districts.

**Results:**

Four main themes emerged from 50 interviews in this study. 1) Many day-to-day traditional medicine procedures pose risks of infection to Traditional Practitioners. 2) TPs’ concepts and understanding of infection are often basic and not evidence based. 3) TPs’ beliefs and attitudes toward infection prevention and control may not prioritize patient-centered care. 4) TPs employ various IPC measures, including some scientifically questionable or incorrect practices.

**Discussion:**

Challenges such as lack of formal medical training and resources hinder effective IPC practices among Traditional Practitioners (TPs). Addressing the gaps in IPC practices among Traditional Practitioners (TPs) is crucial for promoting patient safety and public health in Rwanda. Improving IPC knowledge and practices, providing comprehensive training, and institutionalizing traditional medicine are recommended. Additionally, more research is needed to support the effectiveness and safety of traditional healing practices.

## Introduction

The practice of traditional medicine (TM) represents a diverse array of knowledge, skills, and practices deeply entrenched in indigenous theories, beliefs, and experiences across various cultures for centuries (WHO, 2023). TM fulfills a multifaceted role that includes promoting health, preventing, diagnosing, and treating both physical and mental ailments (WHO, 2023). In regions with limited access to conventional medical services, Traditional Practitioners (TPs) play a pivotal role as primary healthcare providers, offering accessible and culturally relevant healing methods (Tan *et al.*, 2021; Takeyama *et al.*, 2022; Florio *et al.*, 2023).

Recognizing the significant contributions of TM to the advancement of universal health coverage, the World Health Organization (WHO,2023) emphasizes the importance of governmental endorsement and regulation of this practice (WHO, 2024a), (WHO, 2024b). As of 2018, 98 WHO Member States have developed national policies on traditional complementary and alternative medicine (TCAM), with 124 countries implementing specific regulations to ensure the safety, quality, and efficacy of herbal remedies (WHO, 2019). This global effort underscores the commitment to integrating traditional healing practices into mainstream healthcare systems (WHO, 2019).

In many countries, the absence of formal medical training programs or minimum educational requirements for TM has been a historical issue (Mutombo *et al.*, 2023). For instance, in Rwanda, approximately 31% of TPs have not undergone any formal schooling, resulting in a significant variation in the educational backgrounds of TPs (AGA Rwanda, 2011; Beste *et al.*, 2015). Furthermore, there is a lack of regulation and mechanisms to ensure the safety, efficacy, and standardization of traditional remedies and practices (Demeke *et al.*, 2022).

During patient care, TPs unavoidably encounter potentially infectious body fluids and materials. However, TPs often rely on makeshift barriers such as bags, paper, sticks, or no protective measures at all to prevent exposure to blood (Audet *et al.*, 2020). Additionally, the practice of using direct mouth-to-mouth resuscitation has significantly increased their risk of contracting HIV and other infectious diseases (Siziya and Hazemba, 2013). Studies conducted in South Africa and Democratic Republic of Congo have demonstrated that TPs who perform treatments involving incisions and injections are 2.4 times more likely to be HIV positive (Audet *et al.*, 2020; Kyambikwa Bisangamo *et al.*, 2024). These findings underscore the urgent need to enhance infection control practices within traditional medicine settings. In Rwanda, despite the availability of free conventional health services through universal health coverage initiatives, TM remains extensively utilized. Recent data indicated that between 50% to 80% of pregnant women in Rwanda relied on traditional botanical medicine as of 2015 (Tan *et al.*, 2021). These statistics underscore the enduring appeal and trust placed in traditional healing practices among the Rwandan population.

The infection prevention and control (IPC) measures undertaken by TPs in Rwanda have not been thoroughly examined (Moh, 2019. Given Rwanda’s dedication to ensuring the quality and safety of TM practices, this study aimed to elucidate the existing practices, challenges, and gaps in IPC measures among TPs in Rwanda. The findings of this study seek to provide insights that can guide the development of focused interventions aimed at enhancing IPC efforts within the TM sector. Ultimately, these efforts aim to contribute to improved healthcare outcomes and the overall well-being of the Rwandan population.

## Materials and Methods

### Settings

This study was conducted through the Abavuzi Gakondo Rwanda Network (AGA Rwanda), an organization of licensed TPs. The organization has more than 1,000 registered members (AGA Rwanda, 2011). The research was conducted in Musanze, Nyabihu, Muhanga, and Bugesera districts in Rwanda – representing different provinces of Rwanda as well as the districts where most Traditional Practitioners practice (AGA Rwanda, 2011).

### Design

This study utilized a qualitative study design through in-depth interviews.

### Sampling and sample size

Practicing TPs who were from the study sites with at least 6 months of experience were randomly selected, regardless of their AGA Rwanda membership. TPs under the age of 18 were excluded.

### Data collection tool

A semi structured interview guide was developed based on previous literature on similar studies (Saunders *et al.*, 2018). The interview guide was developed in English and then translated to Kinyarwanda, the local language. The questions were pretested and modified based on feedback. The final tool included 10 main questions, with multiple probing questions to guide the discussion

### Data collection

Through the AGA Rwanda, the research team contacted the head of TPs in each district, and subsequently, individual TPs. Appointments were made with the TPs who were interested in participating in the study, according to their preferred time and locations. Written informed consents were acquired after a detailed explanation of the study was provided. Audio recordings were used for participants who agreed to be recorded. For those who declined to be recorded, detailed notes of the interviews were taken by data collectors. All interviews were conducted in private space without the presence of any third party. The interview took about 30 minutes and participants were not given any monetary stipend, except for reimbursing their transportation cost. The study was approved by the University of Global Health Equity Internal Institutional Review Board, reference number 213.

### Data analysis

All analyses were conducted manually. The audio recordings were first transcribed and then translated from Kinyarwanda to English. Thematic analysis was conducted following the six-step approach including familiarization with data, generation of initial codes, creating themes, reviewing themes, and writing final report. Transcripts were read and initial codes were generated inductively first independently then as a team until a code book was developed. Each transcript was coded by two researchers according to the code book, first independently then together as a team, to reinforce the objectivity of findings. The codes were grouped into categories, then used to develop the final themes.

### Ethics Approval

This research was approved by the University of Global Health Equity Institution Review Board (UGHE IRB). After university IRB approval, the research team acquired approval from all participating districts before starting the data collection. Reference number 213.

## Results

In-depth interviews were conducted on 50 TPs, their demographic information was summarized in table 1.

**Table 1:** Demographic information

Sample	Characteristics	N=50
Age	Above 40	27 (54%)
	40 and below	23 (46%)
Sex	Male	26 (52%)
	Female	24 (48%)
Marital Status	Single	9 (18%)
	Married	40 (80%)
	Widow	1 (2%)
Place of work	Rural	33 (66%)
	Urban	17 (34%)
AGA Rwanda Membership	Yes	38 (76%)
	No	12 (24%)
Number of patients per week	Below 10	30 (60%)
	10 and above	20 (40%)

Four main themes emerged from the results. Theme 1: Many day-to-day traditional medicine procedures pose risks of infection to Traditional Practitioners. Theme 2: TPs’ concepts and understanding of infection are often basic and not evidence based. Theme 3: TPs’ beliefs and attitudes toward infection prevention and control may not prioritize patient-centered care. Theme 4: TPs employ various IPC measures, including some scientifically questionable or incorrect practices.

### Theme 1: Many day-to-day traditional medicine procedures pose risks of infection transmission to Traditional Practitioners.

Most traditional healing practices entail transforming herbs, typically from their natural plant state into forms suitable for consumption. During the preparation processes, they may injure themselves and be left with cuts and wounds. One participant mentioned:

*“I got injured [often] especially when preparing medications.” [41-year-old male, urban]*

In addition to medication preparation, traditional healing practices frequently involve direct contact with blood and other bodily fluids. One widely practiced technique is *Indasago*, a form of curettage wherein the skin is incised to release blood. TPs perform *Indasago* for various purposes, often to expel perceived malevolent spirits. However, this procedure exposes healers to their patients’ blood, raising concerns about infection transmission and safety.

*“We use razors [to cut their skin] for patients who are bewitched.” [40-year-old Male, rural]*

*“There are times when you are curetting, you touch the blood or even other contaminated fluids because they are too much and spill on you.” [26-year-old female, rural]*

*“We get in contact with blood when we are treating pharyngitis ‘crude tonsillectomy’.” [51-year-old male, rural]*

They also frequently contact patients’ other body fluids such as saliva, as they examine patients. One of the TPs mentioned that his fingers were bitten by a patient when examining his mouth.

*“I was once injured when I curetted a kid. The patient cannot open his mouth, I place a fork on his tongue so that I can look inside the mouth and see the problem…. He bit my fingers.” [26-year-old female, rural]*

For many Traditional Practitioners, their homes double as their workplaces. Within these spaces, they conduct patient consultations, prepare medications, and perform treatment procedures. In cases of critical illness and deemed unsuitable for discharge, some patients may even stay in the healers’ homes. These healers, however, typically lack spacious accommodations to isolate patients showing signs of communicable diseases, thereby exposing both themselves and their families to the risk of infection.

*“We should have a specific room to consult, but I don’t because I am renting.” [48-year-old female, rural]*

*“Space that we work from is small, making it tough to keep the distance and protect ourselves.” [25-year-old male, urban]*

*“They [patients] are poor and don’t have a home, and I tell them to stay at my place for at least a week until they are well.” [48-year-old female, rural]*

*“I should have 2 toilets; one for me and my family and another for patients so that if they have for instance sexually transmittable disease, they cannot transmit it to my family by sharing the toilet.” [38-year-old male, urban]*

Regardless of whether the logic and rationale behind them are sound, the procedures and practices involved in traditional medicine undoubtedly expose Traditional Practitioners to the risk of infection.

### Theme 2: TPs’ concepts and understanding of infection transmission are often basic and not evidence based.

TPs lacked formal training in IPC. They acquired their IPC knowledge through various informal channels, including radio broadcasts, community-based training, or training sessions offered by nearby modern health facilities. However, their understanding of IPC varied widely, ranging from basic to inaccurate. Some believed infection was solely transmitted through blood exposure, while others were unaware that infection could occur outside of large hospital settings. Overall, our participants’ comprehension of infection transmission was limited to one or two aspects.

*“I clean myself and never touch their blood, how can they affect me?” [38-year-old male, urban]*

*“I cannot contract any disease, because I always wash my hands, and do not touch them excessively.” [48-year-old-female, rural]*

*“Because my hands don’t have any wound when I touch the patient, I do not expect my blood to meet with theirs so they can’t infect me.” (35-year-old male, urban)*

*“Since I do not own a hospital in my house, I do not need gloves, gauzes, face masks.” [35-year-old male, urban]*

Some Traditional Practitioners also attribute communicable diseases to witchcraft. This belief significantly influences their choice of protective measures.

*“Traditional Practitioners often touch blood. Sometimes, when patients have ‘Ibitegano’ [bewitched], we treat in two ways: we apply herbal medicine on the skin and we do ‘Indasago’ (intentional tiny cuts on the skin)” [48-year-old female, rural]*

*“Sometimes, someone who has been bewitched and comes with swollen legs. It can be so severe that the circulation in the legs is blocked by water in the legs. To treat them, you use a razor blade, a new razor blade, and drain the water from the leg, until there is no more.” [80-year-old male, rural]*

Despite variations in knowledge about infections and their transmission, nearly all our participants acknowledged the need for more IPC training. They recognize that not all TPs adhere to the same standards and believe that government-led training initiatives, in collaboration with AGA Rwanda, as well as training sessions facilitated by modern medicine doctors, would significantly enhance their overall IPC knowledge.

*“It is a concern that not all Traditional Practitioners have the same level of knowledge.” [41-year-old male, urban]*

*“We need training on how to prevent and protect ourselves from infectious conditions and how to better take care of our patients… our profession needs to be valued. The government needs to give us regular training.” [80-year-old male, rural]*

*“We are known but they can think about us, train us at advanced level, teach us how we can properly treat patients or incorporate us into working with government.” [26-year-old- female, rural]*

*“…Having literate Traditional Practitioners who have a certain education background and the government to teach and train Traditional Practitioners about this job.” [25-year-old- male, urban]*

*“The training will increase my knowledge on protective measures and how to manage many patients…” [37-year-old- female, rural]*

### Theme 3: TPs’ beliefs and attitudes toward IPC may not prioritize patient-centered care.

A common recurring belief mentioned by many TPs was that they were protected by God or some divine spirit, thus they were immune from harm and diseases.

*“As a Traditional Practitioner, I am already protected, I am already immunized by the spirits.” [80-year-old male, rural]*

*“I think TPs are protected by the God” [60-year-old female, rural]*

While many TPs believed that by protecting themselves, they could also protect their patients from contracting diseases, some were primarily focused on safeguarding their own health. When suspecting that patients were contagious, these healers refrained from direct contact and delegated tasks to others, such as the patients’ caregivers. Additionally, some maintained distance from their patients as a precautionary measure to prevent the transmission of diseases.

*“We first protect ourselves before protecting them…”* *[51-year-old male, rural]*

*“The trainings we attended taught us that we must protect ourselves. The patients might infect you.” [33-year-old female, rural]*

*“There are children with ubuheri (skin rashes) that are ulcerated. You should know how to protect yourself, I let the patient’s mother be the one to clean the wounds. After she finishes cleaning the wounds, you give the medications to the parent to apply on child’s wounds… I try to help the patient while keeping a safe distance, but not making it so obvious that you will keep the connection with the patient” [61-year-old female, rural]*

*“…I move from one patient to another without washing my hands). When I receive a married couple, I understand that they live together, they tend to already have similar conditions.” [45-year-old female, urban]*

Some participants mentioned that when they suspected they were exposed to infection, they would immediately go to the nearest hospital for consultation.

*“If I feel sick, I will go to the hospital to make sure I am ok.” [61-year-old female, rural]*

*“[after I was bitten], I immediately went to the hospital, and they bandaged me.” [26-year-old female, rural]*

### Theme 4: TPs employ a variety of IPC measures, including some practices that are scientifically questionable or incorrect.

Some of the IPC practices by TPs, though simple, were generally appropriate. For example, they prohibit patients from spitting on their working ground area, use separate sets of items for different patients, or simply keeping their hands and area clean.

*“…when they [patients who have coughs] come, I give them a cup or a covered bottle to spit in. So, he is not coughing and spiting on the ground… Where needed, I will give a patient him clean water to drink and soap to wash himself” [61-year-old-female, rural]*

*“We can’t know exactly when an infectious disease is being spread but when patient come in, I first teach them about hygiene.” [38-year-old-male, urban]*

*“Some medications come packaged in cups. When the cups are empty, I wash them and reuse them. I would use new cups each time if I had the means. Patients do not share spoons. For other tools, I wipe them after using them on one patient.” [40-year-old-male, rural]*

*“The set of equipment that I use on one patient is different from what I use on the next patient. If I have 3 patients, each one of them has their own cup and spoon and every equipment is cleaned after use to be reused again later. We do all of this to make sure no patient gets a disease from another patient.” [69-year-old-female, rural]*

Many of our participants recognized the importance of using personal protective equipment (PPE). While gloves and masks were acknowledged as effective, they were infrequently used due to cost constraints. Instead, participants often use alternatives such as street clothes, mosquito nets, and plastic bags as barriers between themselves and their patients to prevent infection transmission.

*“We try to wash them after use, and when they have been used at least two times, that’s when we decide that they are old, and we should throw them and use new ones. Also, when they are torn for the first time, we throw them and use new ones. But it is rare to get torn.” [51-year-old male, rural]*

*“It is very hard to get facemasks to use every day and you cannot reuse them. Facemasks are expensive.” [35-year-old male, rural]*

*“…We put on clothes to protect us from getting infectious conditions.”[80-year-old male, rural]*

*“Most of the time I use a plastic bag. You might also use a cloth on your hands.” [48-year-old female, rural]*

*“These measures do help a lot. We make sure that our workplace is clean, and we also have a mosquito net-like cloth that surrounds our place that keeps all germs away.” [59-year-old-male, rural]*

*“I think the existing measures are enough. This is because when we wear facemasks and drink clean, safe water, we are protecting ourselves. We also keep ourselves away from unprotected sexual intercourse which lead us to acquire any STI.” [69-year-old-female, rural]*

Some Traditional Practitioners reported using certain herbs prophylactically as a form of self-immunization. These herbs are also utilized for treating and immunizing their patients, and in some cases, as post-exposure infection measures. Many of them attribute the effectiveness of these herbs to spiritual remedies.

*“When I treat a patient and they recover, I immunize them. There are those that have been bewitched, those that have been attacked by spirits, those that ingested bewitched foodstuffs. Once they have recovered, we immunize them from ever getting that again.” [80-year-old-male, rural]*

*“There are some medications, just like you use vaccines, we use immunize ourselves so that you have that medicine we call uruganga, is in your body, so that those spirits cannot come into you…” [80-year-old-male, rural]*

The proper disposal of used items is another crucial aspect of IPC. TPs often encounter challenges in managing medical waste, which may include sharp instruments like razor blades or blood-stained clothes. Common disposal methods among our participants include placing these items in their toilets, burning them, or burying them underground.

*“After using gloves, I throw them in toilet and then pour water in.” [66-year-old female, urban]*

*“When you see these biological wastes [faeces] coming out, you tell the patient to go behind the house and put them in a small hole and you cover it with soil.” [66-year-old female, urban]*

*“I use gloves once and throw them in one place, then after they become many, I burn them together.” [37-year-old male, rural]*

Despite implementing various IPC measures, many TPs remain uncertain about their level of protection. Despite employing numerous techniques for self-protection, most still do not feel completely safeguarded.

*“Not 100%, because you may clean a cup and while it is placed on the cupboard, an insect may fall on it because our tools do not come packed from industries directly. We prepared them by ourselves so we can’t trust it 100%. There is some protection since we cleaned it, but it is not 100%.” [38-year-old male, urban]*

*“Not at all. You cannot feel entirely protected while you do not have those gauzes and gloves.” [48-year-old female, rural]*

*“We are not 100% protected, because we do not do enough investigating to learn more.” [40-year-old male, rural]*

## Discussion

Infection prevention and control is crucial not only for individual well-being but also for the broader community’s health. On a personal level, implementing proper IPC measures serves as a shield safeguarding both patients and healthcare providers from potential harm. At the community level, it acts as a bulwark against the spread of infectious diseases, thus playing an indispensable role in promoting public health. In the context of Rwanda, TPs operate in an environment where formal training programs in traditional medicine are nonexistent. Instead, TPs typically acquire their knowledge through self-guided learning or apprenticeships, with no specific academic qualifications required. Despite their invaluable role in healthcare delivery, many TPs possess only a primary or high school education level (AGA Rwanda, 2011). Lacking formal medical training, they rely heavily on alternative sources of information for IPC, notably radio broadcasts, sporadic training sessions conducted by healthcare facilities, and informal apprenticeships within their families or communities (Beste *et al.*, 2015). Our study findings mirror this reality, highlighting the limited understanding of IPC among TPs, with their knowledge often confined to rudimentary principles. Throughout the interviews, participants frequently linked infections primarily to blood transmission, indicative of gaps in their comprehension of broader IPC practices.

When examining TM practices more closely, the widespread use of techniques like *Indasago* becomes evident not only in Rwanda but also across various regions in Africa. TPs in countries such as Mozambique and South Africa employ similar methods utilizing razors and needles for treatments (Audet *et al.*, 2016; Audet *et al.*, 2020; Bangura *et al.*, 2020). However, this practice poses significant risks, with studies estimating lifetime blood exposures among TPs to be as high as 1,758 instances, thereby increasing their susceptibility to blood borne infections such as HIV, HCV, and HBV (Audet *et al.*, 2016). Similar findings have been documented in Mozambique and South Africa, underscoring the prevalence of such risks within traditional medicine communities (Audet *et al.*, 2016; Audet *et al.*, 2020). Additionally, research has revealed a higher prevalence of HIV infection among TPs who are frequently exposed to patients’ bodily fluids like blood, saliva, semen, or vaginal fluids, compared to the general population (Audet *et al.*, 2016; Audet *et al.*, 2020). These findings underscore the urgent need for comprehensive interventions to mitigate the risks associated with traditional healing practices and protect the health and safety of both TPs and their patients.

Some of our participants described performing *Indasago* as a method for dispelling evil spirits, believing them to be the root cause of certain conditions. Putting aside the rationale of such beliefs, these practices inherently involve the use of sharp objects and blood contact. Given that *Indasago* is a routine procedure in their line of work, it’s understandable that their concept of infection primarily revolves around blood transmission, with a few mentioning other body fluids. One notable observation in our study is that almost no participant mentioned the risk of cross-infection. The concept that patients could contract infections from them did not seem to be their primary concern or understanding. However, this oversight can pose significant dangers to patients. Previous studies in South Africa have revealed instances where Traditional Practitioners jeopardized the lives of pregnant mothers due to their lack of proper training (Thipanyane *et al.*, 2022).

Another misconception identified in our study is that IPC measures were deemed less important and critical because TPs were not operating within hospital settings. Similarly, a study in South Africa found that TPs did not grasp the necessity of using PPE to safeguard themselves in their home-practice environments (Audet *et al.*, 2020). Many health belief models and social-cognitive models have demonstrated that knowledge influences beliefs, attitudes, intentions, and behavior (Fabrigar *et al.*, 2006). Building upon this knowledge-attitude-behavior framework, we can better understand the IPC practices of our participants. The most common cleaning method used by TPs was to clean their treatment tools, such as knives, razors, cups, cooking pots, and saucepans, with soap and boiled water, then sun-drying in the open air. Considering that TM is typically provided at the homes of TPs, it is unreasonable to expect them to sterilize their equipment and tools to the same standard as in hospitals. This observation aligns with the understanding that behavior is influenced by knowledge and attitudes. TPs may not have the same level of understanding or access to resources as modern healthcare facilities, leading them to employ cleaning methods that may not meet medical standards but are practical and accessible in their context. Therefore, interventions aimed at improving IPC practices among TPs should consider their specific knowledge, beliefs, and available resources to ensure effective implementation and adherence.

The general recommendations for cleaning and sterilizing surgical instruments involve utilizing autoclaves with appropriate temperature, pressure, and exposure time, while handling them with sterile gloves and forceps (Rn *et al.*, 2021). Additionally, the use of disinfectants such as alcohol and chlorhexidine are recommended (Rn *et al.*, 2021). These aseptic measures, when carried out correctly, play a crucial role in reducing the risk of patients acquiring infections (Sikora and Zahra, 2024). It’s important to recognize that autoclave machines are not commonly found in the households of TPs, and it would indeed be unrealistic to expect them to use such equipment for sterilizing their tools. Additionally, it is concerning that none of the participants mentioned using disinfectants as part of their cleaning procedures. Furthermore, the reuse of gloves due to financial constraints, as mentioned by many participants, indicates a lack of adherence to proper IPC measures.

Proper disposal of medical waste is a critical aspect of IPC. Contaminated sharps and blood-stained materials have the potential to harbor infectious agents, posing significant risks if not disposed of properly. Improper disposal of such items can increase the risk of spreading pathogens through direct contact or contamination of soil and water sources (Attrah *et al.*, 2022). The disposal methods mentioned in this study are concerning as they were mostly inappropriate, such as throwing used gloves in the toilet or burying biological waste in the ground around their houses. It’s likely that TPs may not be aware of the proper disposal methods or mistakenly believe that such practices are acceptable, perhaps due to convenience or a lack of designated disposal containers readily available. These improper disposal methods were not unique to Rwanda but were also reported among TPs in other countries (Audet *et al.*, 2020). Many TPs cited the high cost of gloves and masks as a barrier to proper IPC practices. While financial constraints may have limited their ability to adhere to recommended IPC measures, it’s crucial to address their lack of IPC knowledge to effect meaningful changes.

Interestingly, TPs themselves expressed a desire for more IPC training, indicating a recognition of the importance of improving their practices in this area. Providing medical services by TPs at their homes presents various challenges for IPC. In our study, participants highlighted the need for specific traditional medicine clinics equipped with proper diagnostic and treatment equipment and materials. Similar challenges and suggestions have been raised by TPs in other countries like Ghana (Nyame *et al.*, 2021). One proposed solution to address these challenges is the institutionalization of traditional medicine by setting up dedicated traditional medicine clinics. These clinics could provide a controlled environment where TPs can offer their services using appropriate equipment and facilities, thus improving IPC practices and ensuring the safety of both TPs and their patients. By establishing traditional medicine clinics, authorities can regulate and standardize practices, implement IPC protocols, and provide training and support to TPs. This approach not only addresses the IPC challenges associated with home-based services but also promotes the integration of traditional medicine into formal healthcare systems, enhancing access to safe and effective healthcare services for communities.

 The idea of integrating traditional healing practices into formal healthcare systems has garnered significant attention in the international community, as evidenced by key milestones such as the Alma Atta Declaration of 1978, the WHO resolution in 2000 promoting the role of traditional medicine in health care systems, and initiatives by organizations like the African Union (Kofi-Tsekpo, 2004). In 2004, the WHO Regional Director for Africa appointed a Regional Expert Committee on Traditional Medicine to develop practice guidelines (Kofi-Tsekpo, 2004). Institutionalizing TM has the potential to enhance access to and the quality of care (Coderey, 2021). Governments can play a pivotal role in standardizing services and monitoring their delivery. To achieve this, adequate allocation of human and financial resources, thoughtful program development, effective regulation and quality assurance measures, and robust research and development efforts to generate scientific evidence supporting the effectiveness and safety of traditional healing practices are essential (Fokunang *et al.*, 2011; Heinrich, 2015; Kasilo *et al.*, 2019; Wang *et al.*, 2023).

## Conclusion

This study aimed to investigate the IPC practices of TPs. The results showed a clear need to improve the IPC knowledge and practices of TPs and to enhance their access to and disposal of protective materials. Addressing misconceptions and providing comprehensive training on IPC practices can help mitigate the risks associated with traditional healing practices and promote the safety and well-being of both Traditional Practitioners and their patients. Adopting a qualitative approach, the study explored the experiences of TPs regarding IPC measures, revealing some questionable practices. Moving forward, future studies can assess the risks of TPs and their patients contracting infectious diseases, generating more scientific evidence to inform policy and protocol development in this area.

### Funding

This research was funded by The Royal Society of Tropical Medicine and Hygiene

### Conflict of interest

The authors declare that there is no conflict of interest associated with this study.

### Availability of data

The datasets used and/or analyzed during the current study are available from the corresponding author on reasonable request.

### Consent to participate

Written informed consent was obtained from all participants included in this study.

**List of Abbreviation:**
TPs: Traditional practitioners;
TM: Traditional medicine;
IPC: Infection prevention and control
HIV: Humana Immunodeficiency Virus;
HCV: Hepatitis C Virus;
HBV: Hepatitis B Virus;
TB: Tuberculosis
